# Educating the healthcare workforce of the future: lessons learned from the development and implementation of a ‘Wearables in Healthcare’ course

**DOI:** 10.1038/s41746-023-00964-y

**Published:** 2023-11-22

**Authors:** Matthew P. Ward, J. Scott Malloy, Chris Kannmacher, Steven R. Steinhubl

**Affiliations:** 1 Weldon School of Biomedical Engineering, Purdue University, West Lafayette, IN USA; 2 Div. of Gastroenterology and Hepatology, Indiana University School of Medicine, Indianapolis, IN USA

**Keywords:** Health services, Biomarkers

## Abstract

Digital health technologies will play an ever-increasing role in the future of healthcare. It is crucial that the people who will help make that transformation possible have the evidence-based and hands-on training necessary to address the many challenges ahead. To better prepare the future health workforce with the knowledge necessary to support the re-engineering of healthcare in an equitable, person-centric manner, we developed an experiential learning course—Wearables in Healthcare—for advanced undergraduate and graduate university students. Here we describe the components of that course and the lessons learned to help guide others interested in developing similar courses.

## Introduction

Healthcare lags far behind virtually every other industry when it comes to taking advantage of digital technologies^1^. Despite that current reality, there is near uniform recognition by major global and national health organizations that digital technologies will shape the future of healthcare^2–5^. Framing the full scope of the necessity and the challenge of making that a reality, the WHO states, “Digital health should be an integral part of health priorities and benefit people in a way that is ethical, safe, secure, reliable, equitable and sustainable”^5^.

Wearable sensors can be a key component of a digital transformation of healthcare globally^6–9^. While most of the physiologic measures that wearables track (e.g., heart rate, respiratory rate, and temperature) are not dissimilar from what is currently measured in healthcare, the wearable-derived versions are uniquely different enough to transform them into individualized vital signs^10^. For example, while a person’s heart rate may be measured over a 10–30 s period every few months or years in the medical office setting, wearables provide near-continuous heart rate data, including routine variation during specific types and levels of activity. These kinds of data allow for the establishment of a “healthy baseline” and the detection of unexpected deviations from that baseline, creating a dynamic, vital sign for each individual.

Extracting actionable information from these large, multivariable, and longitudinal data streams comes with substantial challenges. For one, the data are orders of magnitude messier than the well-controlled snapshot of vital signs obtained in the healthcare setting. Separating out the signal from the noise requires data analytic expertise as well as an understanding of the potential health impact of trade-offs between accuracy, latency, and volume.

An understanding of how the use of wearable data can impact individuals and communities is critical to understanding their potential role in healthcare. For example, since wearable data can lead to health concerns and create anxiety, 24/7 bidirectional, individualized communications capabilities are required to address this in a person-centric manner^11,12^. In addition, appreciating how wearables can further exacerbate—rather than help eliminate—existing health disparities if not thoughtfully implemented is especially important. Assuring that wearables’ role in the future of care drives health equity requires knowledge of existing gaps, historical biases, and purposeful, equitable solution design^13^.

In order to better prepare the future health workforce with the knowledge necessary to support the re-engineering of healthcare in an equitable, person-centric manner, we developed an experiential learning course—Wearables in Healthcare—for advanced undergraduate and graduate university students. Here we report the structure, experience, outcomes, and learnings from this course so that other educators can potentially build off our experience and develop similar courses that will help further accelerate the transformation of healthcare.

### Course description

The Wearables in Healthcare course was developed, in part, to directly expose students to (1) the challenges and opportunities surrounding the use of wearable data in healthcare, (2) the origin and meaning of physiologic signal dynamics measured or derived from data collected by wearable sensors, and (3) the skills needed to process and interpret these continuous data streams. To facilitate a hands-on, real-world experience, all students were given a medical-grade wrist sensor to wear throughout the semester, along with access to all of their raw data streams to permit analysis of their own data. This pairing of personal wearable sensors and data, along with directed reading, lectures, and labs, provided students the opportunity to learn first-hand about their unique physiologic responses to routine behaviors and the difficulties in extracting that information in the most meaningful way. Throughout the 16-week semester, the class exposed them to the multiple challenges that need to be overcome to make these technologies more pervasive in healthcare. The learning objectives for the course are listed in Table 1.

**Table 1:** Class learning objectives.

1	Identify key problems to be solved in medical care and understand how wearable sensor technologies might address them
2	Understand, through first-hand experience, the challenges and capabilities of utilizing wearable sensor data to understand individualized physiologic changes in routine daily activities
3	Learn data analytic skills necessary to accurately translate raw wearable sensor data into meaningful physiologic measures
4	Appreciate the value of individual biometrics detectable via wearable sensors in addressing a myriad of health concerns
5	Demonstrate an ability to apply individual biometrics to a specific health need(s)
6	Understand the need and barriers to meaningfully addressing diversity and equity in all aspects of the utilization of wearable sensors in healthcare
7	Appreciate the challenges of maintaining the privacy of health data and preserving security in all aspects of data handling
8	Address the challenges of precision communication in providing health-related information back to users of wearable sensors

### Students

The initial class was made up of 21 students. The majority were biomedical engineering graduate students, with 22% advanced undergrads. Required prerequisites included at least one signal processing class and research ethics training (either as a class or Collaborative Institutional Training Initiative (CITI) training and certification). All had programming experience, with two-thirds describing their proficiency with MATLAB as being at least intermediate. Students also had the option to work with Python.

Forty-four percent of students were female. When asked to self-identify their skin tone, over half were Fitzpatrick type 1 or 2, with the remainder evenly divided between 3 and 5^14^. Only one student had any work history within healthcare and two students described themselves as having extensive personal experience with the healthcare system. One-third of students wore a smartwatch or fitness band almost always prior to the class, whereas 44% never wore one.

### Wearable device

**Fig. 1:**
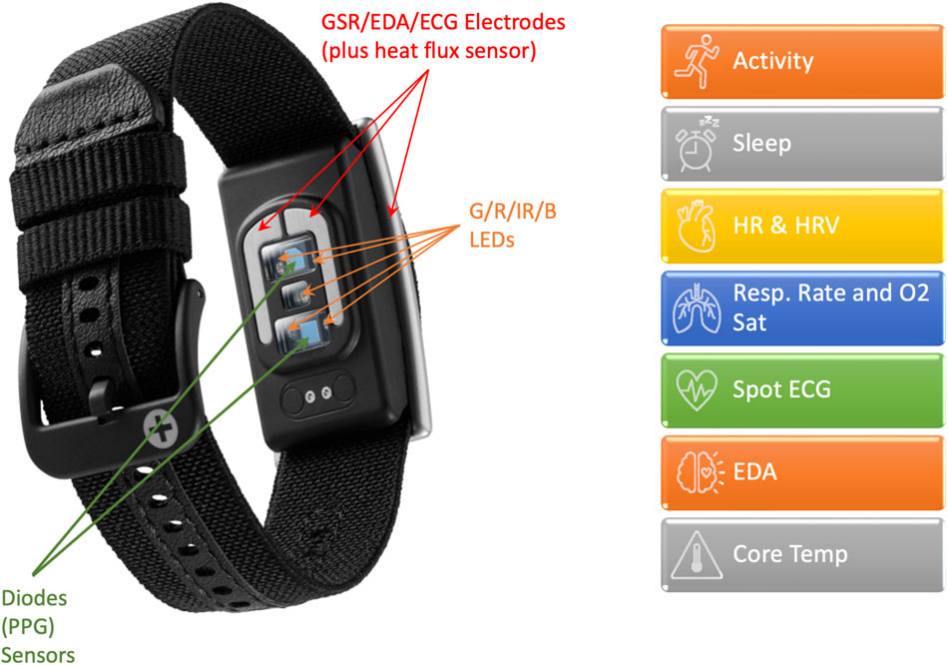
The Corsano 287-2B. Sensors and metrics available from the medical-grade wearable used by students^21^.

All students were given a Corsano 287-2B to use throughout the semester (Corsano Health B.V.). It is a smart band that has been integrated with photoplethysmography (PPG), electrodermal activity (EDA), thermistor, and electrocardiogram sensors for wireless, remote monitoring (Fig. 1). Multiple clinically validated multi-sensor algorithms enable the device to monitor heart rate^15,16^, respiratory rate^17^, sleep staging^18^, arrhythmia detection^19^, and core body temperature^20^, among other biometrics, with their validation studies described in the Instruction Manual^21^. The device achieved CE certification under MDR regulation and is utilizing clinical trial data to apply for FDA certifications in late 2023 or early 2024^22^.

Students wore the device on the wrist and were encouraged to record data often through a variety of situations, including stressful events like presentations, workouts, or other rigorous activities. Assignments were tailored around each student’s own lifestyle to inform them of their responses to events of interest to them. The device connects with Corsano’s phone and web application to collect data without any interference to the user. In accordance with general data protection regulation (GDPR), Corsano removed all student data from the health portal after the semester.

**Fig. 2:**
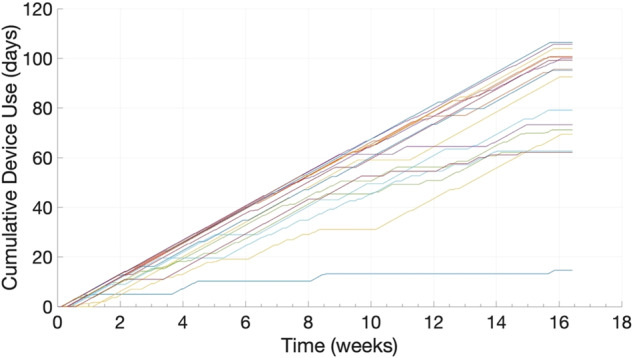
Device usage summary. Individual student cumulative wear time over the duration of the semester (*n* = 18 of 21 total students in the class).

Students on average reported wearing the device 81% of days for at least 4 h and 75% of nights. As shown in Fig. 2, persistent wear was the norm for the majority of students. Most students had no issues wearing the device through regular activities, however, 33% described interference with sports or other behaviors. Two students reported mild skin irritation that created challenges when wearing the device. At nighttime, 41% of students felt that the device was disruptive enough to remove on occasion before or during sleep. Technical difficulties from the Corsano device were limited to some occurrences of insufficient data transfer to the Corsano application and one device malfunctioning, which was replaced.

### Class components

The course was co-taught by two biomedical engineering faculty with different, but complementary expertise spanning data analytics, machine learning, clinical medicine, and digital health technologies in health research and care.

**Fig. 3:**
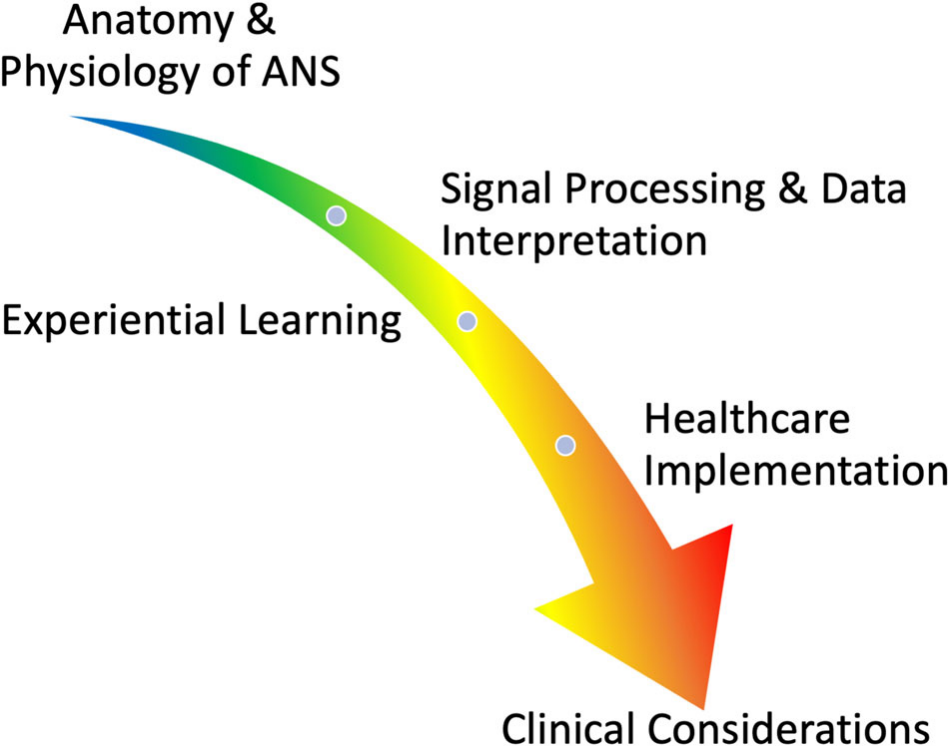
Five focus areas of class sessions. Throughout the semester, students progressively gained the knowledge and skills necessary to appreciate the unique challenges and opportunities in the implementation of wearable sensor technologies in healthcare.

Class sessions were of five general types (Fig. 3). Multiple components of the class were designed to maximize the hands-on learning experience. For example, students (1) learned about the anatomy of the cardiovascular and autonomic nervous system (ANS) and its effector tissues through an extended reality cadaver lab (VH Dissector XR; Touch of Life Technologies, Inc.), (2) were given a challenging pop quiz and asked to analyze their GSR/EDA responses before, during, and after the quiz *after* we told them it was not for a grade, (3) were asked to analyze their physiologic responses during honest versus deceptive play in a card game called Bluff, a popular social game whose object is to get rid of all cards first, most often by deception, (4) learned about the clinical uses of biofeedback therapy (e.g., vagal breathing exercises) for stress management from a licensed pediatric clinical psychologist, and (5) were given the opportunity to explore their physiologic response to public performance through an end-of-semester Karaoke Lab, which was held at a large auditorium at Purdue to simulate a true stage performance.

### Outside of class assignments and testing

Early in the course, work outside of class was primarily focused on required reading assignments that included the technical specifications and user manual of their wearable device, recent peer-reviewed articles covering lecture topics, and specific chapters from the book *Behave: The Biology of Humans at Our Best and Worst*^23^ to provide students with an understanding of the biological processes that influence human behavior. As the semester progressed homework assignments chiefly involved students analyzing their own raw data and writing their own signal processing workflows to extract physiologic and behavior (e.g., activity) information from the raw data streams. These assignments culminated in an end-of-semester project that required students to generate code and analyze their physiologic response to a mild-moderate stressful event, like watching a scary movie or taking an exam. Students were required to analyze all available physiologic data from their device, including skin temperature, GSR/EDA, heart rate derivation from the PPG signal, HRV analysis in the time and frequency domain, respiration rate, and activity levels. Students were asked to quantify and describe the meaning of differences in computed biometrics from the stressful period in comparison to the same period of time on a different day when the mild-moderately stressor was not present.

The course only included one multiple-choice, in-class, midterm exam and one final essay exam that could be completed at home. The midterm exam included pertinent anatomy & physiology topics covered in class lectures and wearables-associated health information. For the take-home final essay, students were allowed to use large language models (LLMs) such as ChatGPT 3.5, which was freely available to all students. The test required that the students answer three questions, all dealing with hypothetical scenarios that raised important ethical questions regarding wearables and the data they produce. Because the important role of artificial intelligence, especially LLMs, was frequently discussed in class as having a potentially important role in healthcare communications around wearable data, its use as a tool to aid students was deemed appropriate. The essay grading rubric emphasized originality in responses. Students were asked at the end of the essay to state whether they took advantage of LLMs to aid their responses, plus their thoughts on using it. All but two students used an LLM, with the two who did not, noting that doing so didn’t feel ethically correct. Feelings were equally mixed as to how helpful students found it, as were opinions as to whether LLMs help or hinder learning for students.

### Student experience

Students in this course were responsible for collecting and analyzing the data recorded from their Corsano devices. This process involved downloading datasets from Corsano’s user portal and utilizing MATLAB (or Python) to perform various analyses. An important aspect of the course was collaboration on homework and projects, which involved sharing data if the students felt comfortable doing so or simply sharing programming tips. At the completion of the course, 94% of students had been comfortable with voluntarily sharing their datasets, but one student mentioned, “I didn’t love the idea of giving out my data”.

The lectures generally covered three main categories: data analytics & interpretation, physiology & anatomy, and wearable data in health. Overall, students felt the amount of instruction on each topic was “just right” (data analytics: 69%, physiology and anatomy: 83%, wearable data in health: 89%). However, 27% of students felt that there was too little data analytics instruction, and 22% felt there was too little physiology and anatomy instruction.

Ninety-four percent of students felt that analyzing their own data enhanced their learning experience and 75% felt that the course enhanced their understanding of human physiology. However, six students stated that at some point throughout the semester, looking at their data raised some concerns about their health. Of these students, three resolved their concerns by talking to friends/family or on their own, but three students felt they never resolved their concerns.

Students were asked if the course experience had changed their willingness to wear a sensor. Seventy-two percent stated it would not change their pre-class wearables behavior, with the majority continuing to wear one daily. For the remaining 28%, their experience in the class inspired them to start wearing a device daily. Importantly, when asked if they would recommend the class to other students, 100% answered “yes.”

### Lessons learned and future direction

The first implementation of this course was in most ways a success. However, there is room for improvement and refinement based on the lessons learned from implementation and from valuable student feedback. Many of these take-home points have been summarized in Table 2 as key considerations for others who may wish to implement a similar course.

**Table 2:** Key considerations for implementation of similar courses.

Key consideration	Student perspective	Faculty learnings
Students have different lived experiences and skillsets pre-class	Two of the 18 respondents did not feel they had an adequate background to get the most out of the class due to inadequate data analytics/MATLAB experience	It is important to be more explicit in prerequisite requirements, especially for programming in MATLAB or Python, and to provide additional resources to ensure no students fall behind
Students may have differing attitudes and beliefs around data privacy and sharing	Despite the zero-tolerance policy for compelling others to share their data, at least one student was concerned about the perception of covert pressure to share personal data	Providing a deidentified group data set for all students to work with as a team would eliminate both stated and unstated student privacy concerns for team-based labs/assignments
A misunderstanding of personal physiologic data may raise health concerns	For some students, their sensor data caused concerns – many that were verbalized included concerns about sleep duration and heart rate	Be very explicit, from the start, that for some people, wearable data can cause anxiety, and spend time in class discussing these types of challenges to adoption/use of DHTs While having a physician instructor was of some help, some students might not feel comfortable approaching an instructor
Scaling up the size of the class without diminishing individual attention	Even the present size (21 students) was too large to enable the one-on-one instruction some students needed	Use teaching assistants when permitted Better formalize working groups through the semester for peer-to-peer help
Affordability of wearable devices	Requiring students to buy their own device would limit participation in the class and perhaps the quality of learning	This type of class would work well with corporate partnerships to either make devices available at no cost to students or at a substantially discounted rate

The course is intended to serve upper-level undergraduate and graduate students from a variety of backgrounds and lived experiences. While we required prerequisite experience with signal processing, many students still struggled to use MATLAB or Python for this purpose at the desired competency level. To help “level the playing field,” we decided to provide a sample code for all assignments that all students were free to use or modify for their assignments. Class time was used to explain the theory and operation of various signal processing and data handling techniques that are common to the discipline. Students were required to cite any borrowed code and to explain what the code does in their own words for full credit on assignments. Providing the code to students did not appear to detract from their understanding of how to work with these physiologic data streams, but rather freed up their minds to focus more on the underlying anatomy/physiology that drives changes in these data streams and what it all means for personal health management.

While this initial class offering was focused on biomedical engineering students, we anticipate that many other students with an interest in health-related careers would also gain value from a similar offering. To adapt the course for a more clinical audience, we would recommend the following modifications:

- Consider purchasing less-expensive wearable devices that do not necessarily provide access to raw data streams. This would allow new instructors to offer their courses to more students with the same budget.
- Consider replacing the programming-related components of the course with labs that investigate how to identify and/or aid the diagnosis of various pathologies via the analysis of trends in individual data over time or in response to a particular controlled circumstance.
- For nursing students, develop labs to train students to identify signs of distress or pain in their patients by learning how to identify the signatures of acute stress and pain from the dynamics of various biometrics over short (e.g., seconds to minutes) to long (hours to days) timescales.

In the first class of the semester, we spent time discussing how to handle data privacy throughout the term. Students were instructed to never compel anyone else to share their data for any reason, to respect everyone’s decisions without question, and to treat any data that might be voluntarily shared with them as strictly confidential with no permissible use outside of the original purpose for sharing it. Students were encouraged to use this class as an opportunity to learn about themselves and the potential value of wearable sensor-derived biometrics in managing individual health, so analyzing their own data for assignments was encouraged. This pedagogical decision gave the class an opportunity to understand the vast differences in physiology among us, along with the striking similarities when interpreted from the perspective of interacting physiological systems common to us all. Students were told that they could ask the instructors (without question or penalty) for a sample data set at any time if they did not feel comfortable analyzing their own data for assignments. Despite these guarantees, at least one student reported not feeling comfortable with the system, which highlights the need for more periodic reminders that generic datasets are available for students who do not want to share any analysis of their own data.

The notion of (mis)understanding individual health within the confines of population-based metrics and standards served as a recurring theme throughout the semester. We designed the bulk of the course content to help students to understand the value of interpreting changes to individual health with respect to their own trends and norms as opposed to population-derived standards. However, we did not adequately emphasize to students that seeing one’s own data may trigger anxiety. We recommend explaining the types of anxiety that some students may experience and providing appropriate resources to them, including stopping the use of their watch altogether until an alternative solution is found with guidance from the instructors.

## Conclusion

This is the first class that we are aware of to offer an experiential learning opportunity for wearables in healthcare to university students. While we identified multiple areas for future improvement, a valuable measure of the success of this initial offering was that 100% of the students stated they would recommend the class to other students. With digital health technologies poised to play a central role in the future of healthcare, it is crucial that the people who will help make that transformation possible have the evidence-based and hands-on training necessary to address the many challenges ahead in an informed manner as possible. We hope that our experience can inspire others across the globe to improve upon and further expand on our early learnings to help more rapidly accelerate the transformation of healthcare “…that is ethical, safe, secure, reliable, equitable and sustainable”^5^.
